# Innovative approaches in the treatment-resistant depression: exploring different therapeutic pathways

**DOI:** 10.1007/s11357-025-01615-8

**Published:** 2025-03-25

**Authors:** Anna Łysik, Katarzyna Logoń, Aleksandra Szczygieł, Julia Wołoszczak, Martyna Wrześniewska, Jerzy Leszek

**Affiliations:** 1 Faculty of Medicine, Wroclaw Medical University, Wyb. L. Pasteura 10, 50-367 Wrocław, Poland; 2 Department and Clinic of Psychiatry, Wroclaw Medical University, Wyb. L. Pasteura 10, 50-367 Wrocław, Poland

**Keywords:** Depression, Electroconvulsive therapy, Transcranial magnetic stimulation, Deep brain stimulation, Vagus nerve stimulation, Ketamine

## Abstract

Treatment-resistant depression (TRD) remains a vital challenge in psychiatry, affecting a significant number of patients with major depressive disorder. Current pharmacological approaches often do not provide sufficient therapeutic results, prompting the need for innovative treatments. This review summarizes recent advances in TRD management, including non-pharmacological therapies such as transcranial magnetic stimulation, deep brain stimulation, electroconvulsive therapy, and vagus nerve stimulation, and describes their mechanisms of action. Novel pharmacotherapies, particularly glutamatergic modulators like ketamine and esketamine, have shown promising results with esketamine being available to eligible patients in Poland since 2023 within a drug program. Electroconvulsive therapy remains an effective treatment for TRD, usually with small side effects mainly including transient memory impairment, headache, or cardiovascular changes. Transcranial magnetic stimulation is a non-invasive procedure with proven efficacy; therefore several psychiatric organizations recommend it as a treatment option for major depressive disorder in their clinical guidelines. Deep brain stimulation is a relatively new treatment modality for TRD, with its primary risk being associated with the required neurosurgical procedure. Vagus nerve stimulation seems to be a promising adjunctive treatment for TRD, showing significant improvements in depressive symptoms, especially at higher electrical doses but with no side effects. While these treatments appear to have potential, personalized approaches are crucial for optimizing outcomes. Future research should focus on refining the techniques, improving safety profiles, and validating the long-term efficacy.

## Introduction

Major depressive disorder (MDD), a multifactorial mental disease, is one of the leading causes of disability worldwide—currently, approximately 322 million people, or 4.4% of the global population, are living with depression. It most commonly affects women, and its prevalence peaks in late adulthood (after the age of 55). The increasing incidence of depression is reflected in an 18.4% increase in the total estimated number of people living with MDD between 2005 and 2015 by WHO [1].

The therapy of patients suffering from depression presents numerous challenges. Clinical data indicates that the MDD overall cumulative remission rate is around 67% and higher relapse rates are observed in individuals who require more treatment steps [2]. Additionally, approximately 30% of MDD patients are unresponsive to at least two antidepressant regimens of adequate dose and duration and thus meet the clinical criteria for treatment-resistant depression (TRD) [3]. Despite extensive research, the causes and factors influencing TRD are still unclear; internal physiological factors and genetic variability among patients may contribute to the lack of recovery and symptom remission. Currently, the scientifically supported theory suggests that the genes active in patients with TRD are linked to glutamatergic and monoaminergic neurotransmission, along with synaptic plasticity [3].

**Fig. 1:**
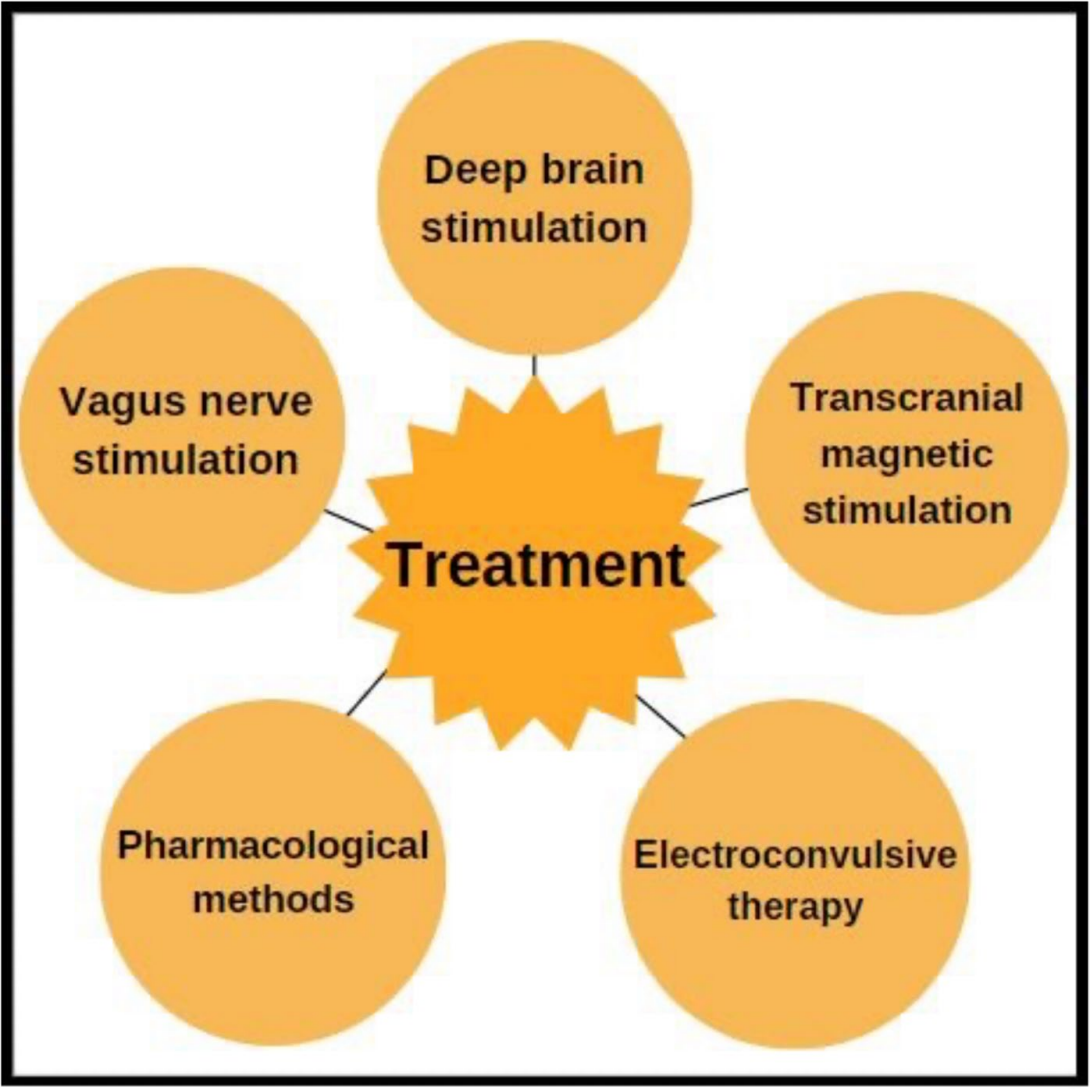
New therapeutic methods used in TRD treatment, 3

Due to the challenging treatment of TRD, new promising therapies have emerged worldwide, including transcranial magnetic stimulation (TMS), deep brain stimulation (DBS), electroconvulsive therapy (ECT), vagus nerve stimulation (VNS), and use of pharmacological methods (Fig. 1).

In this review, we aim to summarize and highlight the latest approaches for TRD treatment and emphasize areas requiring further research in this field.

## Pharmacological approaches

The main pharmacological strategies for TRD include optimizing the dose and time of taking the antidepressant, switching to another antidepressant, and combining antidepressants and augmentation [4]. Augmentation involves adding a second medication that is not an antidepressant to a first-line pharmacotherapeutic treatment. The most commonly used drugs are lithium, lamotrigine, or second-generation antipsychotics.

In recent years, there has been growing interest in the use of novel glutamatergic modulators, such as intravenous racemic (*R*,*S*)-ketamine and its *S*-enantiomer, intranasal esketamine, for the treatment of depression. Their mechanism of action is complex, including multiple neurotransmitter systems, such as the opioidergic, monoaminergic, glutamatergic, and muscarinic systems, as well as substance P and sigma receptors [5]. Analgesic and anesthetic action mainly stem from *N*-methyl-d-aspartate (NMDA) receptor inhibition. The anti-depressive effect may result from HCN1 channel inhibition, d-serine transport inhibition, or *α*7 nicotinic acetylcholine receptor blockage [5].

The first double-blind human study of ketamine was conducted in 2000 [6]. Seven patients with MDD received a single intravenous infusion of ketamine (0.5 mg/kg). The improvement in depressive symptoms was noted within 72 h and lasted 1 to 2 weeks. In subsequent years, studies and meta-analyses have also demonstrated its effectiveness. A 2022 meta-analysis found a notable mean antidepressant effect of ketamine, with the response varying considerably among patients [7]. Less frequent remission was observed in the more treatment-resistant cases. Furthermore, the study determined that the therapeutic effect stays consistent with repeated treatments. A 2023 meta-analysis concluded that both ketamine and esketamine are significantly more effective than placebo [8]. The efficacy of intravenous racemic ketamine was shown to be higher than that of intranasal esketamine. In terms of tolerability, intravenous racemic ketamine did not differ significantly from placebo, whereas esketamine was less tolerable than placebo. A recent 2024 meta-analysis showed that intravenous ketamine may be efficacious at doses as low as 0.2 mg/kg, with increasing dose response at 0.5 mg/kg [9]. Doses exceeding 0.5 mg/kg did not result in a greater treatment response. Esketamine was more efficacious in 56 and 84 mg doses than in 28 mg doses. While targeting a dose of 84 mg, the main consideration has been tolerability.

Esketamine, administered in medically supervised healthcare settings, was approved by the FDA in 2019 as an adjunctive therapy for TRD in adults [10]. Ketamine is FDA-approved only for anesthetic purposes but not for the treatment of psychiatric diseases. In Poland, the drug program for treating TRD with esketamine began in 2023 [11]. It is available to patients aged 18 to 75 years who suffer from recurrent depressive disorders and do not respond to standard antidepressant medications. Eligibility for the program requires strict adherence to specific criteria.

Another substance that has recently gained interest is psilocybin—the major psychoactive alkaloid of some species of mushrooms. Several clinical trials have shown psilocybin’s efficacy in reducing symptoms of depression; however, the majority of research has been limited to small trials of patients with TRD [12–15]. A 2022 double-blind clinical trial investigated the efficacy of psilocybin at a single dose of 25 mg, 10 mg, or 1 mg (control), along with psychological support [14]. Only a dose of 25 mg significantly reduced depression scores compared to a 1-mg dose over a period of 3 weeks, although it was associated with adverse effects, such as headache, nausea, dizziness, suicidal ideation, and behavior or self-injury. A recent 2024 meta-analysis showed a notable improvement in depression scores with psilocybin compared to comparator treatments [16]. Analyses indicated that individuals with secondary depression, older age, and prior psychedelic use experienced greater improvement. While all studies had a low risk of bias, high heterogeneity lowered the certainty of evidence rating.

As inflammation is increasingly considered to play a role in TRD, cyclooxygenase-2 inhibitors (COX-2 inhibitors) were initially the emphasis of anti-inflammatory research in TRD as augmentation options. The 2022 meta-analysis concluded that celecoxib has an antidepressant effect, although heterogeneity in the studies was observed, primarily due to differences in depression type. Despite evidence of publication bias, the analyses confirmed the reliability of the findings [17]. Furthermore, a tumor necrosis factor antagonist, infliximab, has been examined in TRD patients [18]. A meta-analysis revealed that TNF antagonism does not have generalized efficacy in TRD; however, it was effective in patients with increased inflammatory gene expression, such as TNF and C-reactive protein.

**Fig. 2:**
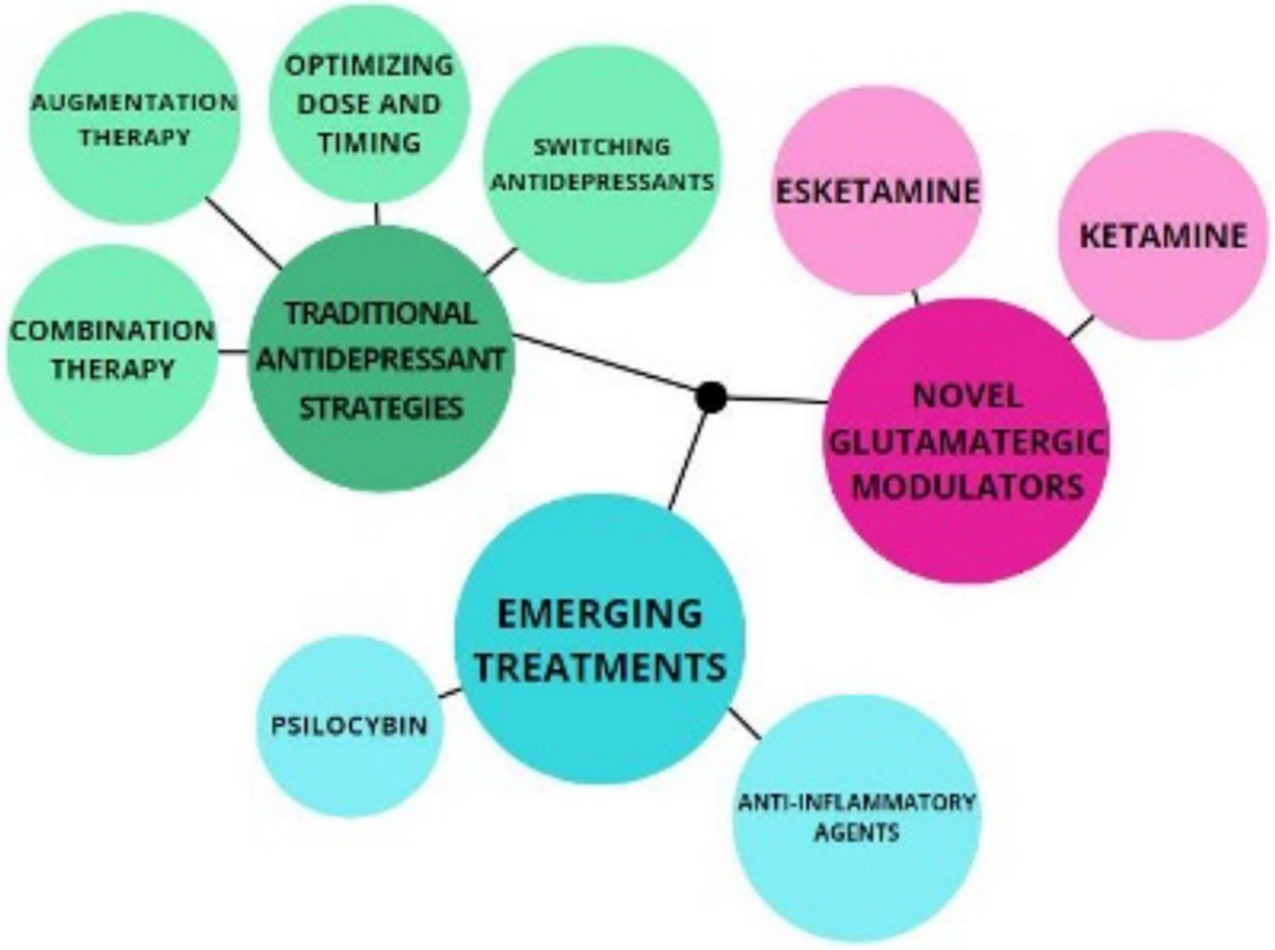
Pharmacological strategies for TRD, 6

Pharmacological strategies used for drug-resistant depression treatment consist of three main areas including traditional antidepressant treatment, emerging treatments, and novel glutamatergic modulators (Fig. 2).

## Electroconvulsive therapy

ECT represents a well-established and cost-effective therapeutic intervention in the management of TRD. The method is widely acknowledged as the most effective acute treatment for severe mood and psychotic disorders and has maintained a prominent position in clinical practice for over 75 years [19].

The efficacy of ECT is thought to be based on four main theories related to neurotransmission, neuroendocrine function, anticonvulsant properties, and neurotropic effects. ECT is responsible for enhancing the availability of neurotransmitters, as well as pituitary and hypothalamic neurohormones; it increases receptor sensitivity and improves neurotransmission, which also contributes to anticonvulsant effects. The neurotrophic theory suggests that ECT may have a positive impact by promoting neurogenesis and increasing neurotrophic signaling in the brain [19].

The procedure is conducted under general anesthesia and requires a thorough evaluation of the patient’s health and risk factors, as well as a comprehensive analysis of taken medications, as it may influence the risk of adverse effects stemming from ECT itself and from the analgosedation performed both psychiatric and ones taken due to comorbidities [20]. The National Network of Depression Centers Task Group on ECT recommended capturing treatment types acute and maintenance. Acute type is used to achieve a clinical response during clinically acute episodes of illness, usually involving 6–12 treatments, 2–3 times per week. Maintenance ECT, however, entails the application of successive treatments with a typical 1-week interval between each session. This approach aims to consolidate a positive response to acute treatment and extend the period of remission [21]. The study by Ross et al. aims to describe the cost-effectiveness of the procedure; research shows possible optimizing the effectiveness of therapy with the inclusion of ECT after two unsuccessful attempts with medication or psychotherapy [22, 23]. It can also be considered a primary treatment for depression with life-threatening psychotic or suicidal features [24]. Expanding the utilization of ECT by offering it earlier in the treatment of depression could significantly enhance outcomes for this group of patients.

The use of the most effective electrical dosage via stimulus titration for the individual seizure threshold, treatment frequency, and device parameters allows for increased efficacy, minimizes adverse reactions, and personalizes therapy to a greater extent [25–28]. In contemporary ECT practice, three electrode placements are commonly used: right unilateral (RUL), bilateral (BL), and bifrontal (BF). RUL electrode placement is as effective for MDD as BL ECT and is associated with fewer cognitive side effects, especially when short-term cognitive deficits are greater with BL placement [27, 28]

Electrical stimulus parameters such as pulse width, pulse frequency, stimulus duration, and pulse amplitude are included to ensure a sufficient understanding of the dosage delivered by the various ECT devices currently in use. The proper selection of parameters and placements of electrodes are the subject of many studies [25–27, 29–31]. The study by Sackheim et al. presents an analysis of the relationship between electrode placement, applied dose, and pulse length. It highlights the advantages of ultra-brief pulses (0.3 ms) over traditional brief pulses (1.5 ms), particularly in reducing acute side effects and minimizing both short- and long-term cognitive [27]. RUL ECT compared to BL ECT in traditional high-dose pulses was also distinguished by a reduction in side effects with similar efficacy, while ultra-brief RUL ECT with high dose relative to threshold compared to traditional brief pulses recorded much better therapeutic results, but without affecting efficacy as expressed by a reduction in relapses [27]. In randomized and blinded clinical trials, it is suggested that low-amplitude seizure therapy has faster reorientation and possibly lower cognitive side effects compared to standard ultra-brief RUL ECT [30]. The frequency evidence suggests that outcomes are comparable between the three-times-per-week and twice-a-week schedules and that a three-times-per-week schedule may produce results slightly more quickly but cause somewhat more cognitive impairment [32, 33].

The effectiveness of therapy is assessed using structured evaluation scales. The assessment should be done before starting therapy and then at weekly intervals to monitor response to treatment. The scales available to assess symptoms of depression are the Hamilton Depression Rating Scale or the Montgomery-Åsberg Depression Rating Scale [34, 35]. Designed to assess cognitive function are the Mini-Mental State Examination or the Montreal Cognitive Assessment [36, 37]. Scales usually performed at the beginning and end of treatment are designed to assess cognitive function; the need for more frequent assessment occurs when cognitive impairment is evident.

Among adverse effects, mostly acute cognitive impairments subsiding during 3 days to 2 weeks post-ECT are recognized, like anterograde and retrograde amnesia and executive functioning impairments. Processing speed, global cognition, and spatial problem-solving may also present minor deficits. Postictal confusional states presented with anxiety and disorientation may occur in up to 20% of patients after ECT treatment [38, 39].

Cardiovascular events are mainly present in patients with multiple risk factors and previously documented cardiac disease. They may experience temporary increased blood pressure, increased heart rate, or arrhythmias with ECG abnormalities. In rare cases, patients experience transient asystole, cardiomyopathy, or myocardial infarction [40–42]. Musculoskeletal side effects caused mostly by anesthetics may manifest as apnea and further respiratory failure, myalgias, and headaches [40, 43].

A majority of patients with MDD show a significant clinical response to acute treatment, but up to 50% do not experience full remission, and between 33 and 50% may experience relapse, even with maintenance therapies [21]. With an average open‐label remission rate of 48% in non‐psychotic depression, efficacy may be higher in individuals with psychotic depression [38]. The ECT group demonstrated a significantly lower mortality rate compared to both the inadequately treated with antidepressants and non-treatment groups. Furthermore, the ECT group had significantly fewer suicide attempts than the antidepressant treatment group, regardless of previous suicide attempts [39].

## Transcranial magnetic stimulation

Prefrontal transcranial magnetic stimulation is a treatment method which uses a device to generate magnetic fields in order to stimulate particular areas of the cerebral cortex. The stimulation is a non-invasive method modulating the activity of neurons. The magnetic stimuli penetrate the cranium and induce an eddy current underneath the scalp, which affects the neuronal membranes, resulting in the generation of an action potential or either an excitatory or inhibitory postsynaptic potential [44]. The magnetic stimuli cross the barriers between the scalp surface and the cortex and get converted to electrical ones on the surface of the brain which induces changes in brain activity. The magnetic field reaches the skull via a circular coil placed directly on the scalp. Repetitive TMS (rTMS) is used in order to evoke long-lasting cortex activity changes, while single-pulse TMS helps to observe brain functioning [40].

TMS is approved to be used as a treatment for drug-resistant MDD. This method is able to stimulate particular regions of the brain depending on their depth and impedance; therefore, high-frequency TMS is applied to the left dorsolateral prefrontal cortex (DLPFC), whereas other regions involved in depression cannot be reached by the stimulation. Those unreachable yet important depression pathophysiology regions include the hippocampus, subgenual anterior cingulate cortex, and other limbic structures [41].

Approved protocols for TMS treatment include either low-frequency stimulation on the right DLPFC as well as high-frequency stimulation on the left DLPFC or combining both of them together. The right DLPFC’s hyperactivity along with the left one’s hypoactivity is believed to be partly responsible for depression development, hence the trials to change their activity using low-frequency stimulation responsible for neural inhibition and high-frequency one which induces neural excitation [42]. Stimulating both right and left DLPFC simultaneously is predicted to result in better outcomes and optimization in depression treatment than sequential stimulation. Such protocols may be performed using an H1 coil which produces a magnetic field covering the prefrontal cortex with bilateral stimulation [43].

However, not only does TMS change brain activity in selectively stimulated areas, but it also alters neuronal activation in numerous regions such as the precentral gyrus and posterior cingulate in the right hemisphere as well as the inferior frontal gyrus and middle frontal gyrus in the left one. There are even more observed areas of neuronal activity alterations impacted by TMS shown in task-related or resting-state studies which need to be followed by further research in order to explain their possible functional consequences [45].

The efficacy of TMS treatment depends on many factors including patient-, procedure-, and illness-related ones. However, the TMS response is challenging to predict because there is no specific factor of this method’s efficacy; therefore, many of them should be taken into consideration to estimate the patient’s response. Among patient-related factors, age appears to be the most significant predictor of TMS therapy outcomes. The prefrontal atrophy observed among older patients seems to be linked to their decreased response to TMS treatment in comparison to younger patients; hence, the younger ones might benefit from a fewer number of stimulating pulses per session to obtain more satisfaction [46]. Other variables affecting TMS treatment efficacy could be the severity of depression episodes, its nature, and its symptoms such as a sense of guilt and depressed mood. Those last two mentioned symptoms seem to be negative predictors [47]. The TMS procedure itself is another source of factors affecting the response to the treatment. Due to some anatomical variations and different activity of targeted cortex regions among patients, the standard technique of TMS may not effectively proceed. In order to solve the anatomical variability problem, connectivity-based targeting could be considered [41].

The efficacy and safety of TMS have been confirmed many times; hence, several psychiatric organizations have included this method as a recommended treatment for MDD in their guidelines. Clinical features of patients receiving TMS therapy include moderate to severe treatment resistance in the current episode, a recurrent course of depression, and moderate to severe illness severity. TMS is also recommended for patients previously treated with a described method followed by positive outcomes who suffer from recurrence of the illness. Another clinical recommendation for using TMS is continuation or maintenance treatment for patients benefiting from this method as well as reintroducing therapy among patients with the following episode of depression who had previously responded to TMS [40]. According to two large randomized controlled trials left prefrontal rTMS should be conducted daily for at least 3 and up to 6 weeks among patients who had failed previous antidepressant trials. Partial responders who completed a 6-week treatment course may attend the extended course with either the same or altered protocol [48, 49].

According to recent studies, patients treated with rTMS respond with time delay rather than immediately [50]. The effects may persist for some period, approximately 5 months before the illness relapses. In order to decrease relapse risk, maintenance protocols should be carried on. Nevertheless, no common consensus about maintenance protocols exists for MDD. Pursuant to some research conclusions it is crucial to maintain the TMS protocol for about 6 months following the acute treatment phase considering remission sustainment [51].

In comparison to ECT, TMS therapy seems to be slightly less effective considering depressive symptoms reduction. However, due to several limitations and lots of requirements related to ECT, TMS treatment’s preponderance arises from its less invasive procedure and lack of adverse effects. Both methods demonstrate a significant therapeutic effect compared to placebo, yet neither of them presents strong evidence of their efficacy and safety. Thus, the choice between them should be based on medical and subjective rationale [52].

## Deep brain stimulation

DBS is a non-pharmacological therapeutic option consisting of the implantation of electrodes in specific brain regions, delivering continuous electrical stimulation to modulate dysfunctional neural circuits and promote neuroplasticity. It was initially used for several neurological conditions such as Parkinson’s disease, dystonia, or essential tremor but its applications have expanded and now include psychiatric conditions such as MDD [53, 54]. However, the exact mechanisms by which DBS exerts its antidepressant effects remain unclear, with research focusing on changes in neurotransmitter release, neurotrophic mechanisms, neuroinflammation, and intracellular signaling processes [55].

In TRD, DBS is a relatively new treatment method and should be regarded as an experimental therapy. Several brain areas associated with mood regulation and affective processing are used as DBS targets, with the choice of target being critical for efficacy. Most often targeted neuronal structures are the subcallosal cingulate cortex (Brodmann area 25) and ventral capsule/ventral striatum, and although the results are limited, the outcomes of studies on these certain DBS targets are promising due to relatively high response and remission rates among patients treated with DBS [56, 57]. Other potential targets needing further research include the anterior limb of the internal capsule, nucleus accumbens, epidural prefrontal cortical, medial forebrain bundle, lateral habenula, inferior thalamic peduncle, supero-lateral branch of the medial forebrain bundle, and posterior gyrus rectus [58].

The efficacy of DBS treatment in TRD is the subject of several meta-analyses and remains a topic of ongoing studies [59, 60]. There are suggestions that the DBS method can have sustained antidepressant effects but factors influencing treatment outcomes include the specific brain targets, patient selection criteria, and stimulation parameters. Personalized and symptom-based approaches to electrode placement and stimulation settings may increase therapeutic outcomes [61]. For instance, individualized tractography targeting to guide electrode positioning is a promising method for enhancing response rates among patients with TRD [62].

Findings suggest that DBS is a promising method that can lead to significant improvement in depressive symptoms with a response rate of about 60% and a remission rate of about 30% in patients with TRD [63]. However, the outcomes of studies have not been uniformly positive. For instance, the randomized sham-controlled trial (Holtzheimer et al., 2017) found that there were no significant differences between active and sham groups after 6 months of DBS treatment and highlighted that future studies are needed and factors such as patient selection and stimulation parameters should be investigated [56]. Additionally, suicide and suicidal ideation have been reported among patients receiving DBS treatment. Data from the meta-analysis revealed that the median rate of suicidal attempts was 16.7% and the rate of suicide was 4.8%. These statistics indicate that DBS is not a completely safe method, and problems related to suicide should always be taken into consideration [58].

There are findings indicating that females with TRD exhibit higher response rates to DBS treatment than males. This gender difference in treatment outcomes needs a comprehensive investigation to uncover the underlying factors. One possible explanation could be the variations in brain connectivity between sexes, known as connectomic sexual dimorphism, which may influence the efficacy of DBS. Additionally, differences in depression phenotypes between males and females could play a role, with females potentially exhibiting a subtype of depression that responds more favorably to DBS [63].

The variability in DBS trial results for TRD underscores the need for better study designs and a more personalized approach that would be crucial for improving DBS effectiveness. Better targeting through individualized tractography has shown promise in increasing response and remission rates [62]. Advanced imaging techniques, such as diffusion tensor imaging (DTI), allow for precise mapping of white matter tracts associated with targeted brain regions and understanding which parts of the brain are influenced by modulation [64]. Biomarkers that would allow the phenotyping of TRD into distinct subtypes, predicting the response, and better target selection hold the potential for personalizing TRD treatment but need more validation [65]. Combining DBS with therapies like pharmacotherapy or cognitive-behavioral therapy (CBT) may further improve outcomes. Additionally, momentary assessment techniques like the experience sampling method can offer detailed mood tracking, aiding clinical decision-making. Overall, advancing DBS for TRD involves integrating clinical, neurophysiological, and imaging data to tailor treatments and optimize patient outcomes [61].

DBS is a relatively safe procedure. No cognitive decline was observed, and the findings even suggest that DBS may have slight positive effects on cognitive functioning in TRD patients [66]. As a surgical procedure, it carries risks such as infection, pain, and seizure. Neuropsychiatric side effects, such as a relative increase in depression symptom severity and irritation have been reported [67]. Long-term safety data are encouraging, with many patients maintaining benefits for years without adverse effects [68].

Deep brain stimulation holds significant promise for individuals suffering from TRD, offering hope where conventional therapies have failed. While challenges remain, particularly in understanding the precise mechanisms and optimizing treatment parameters, current evidence highlights the considerable potential of DBS as a revolutionary treatment for TRD. Future research aimed at refining targeting techniques, personalizing treatment, and developing adaptive closed-loop systems will be essential in realizing the full therapeutic potential of DBS.

## Vagus nerve stimulation

While vagus NS is currently widely used in the treatment of pharmacoresistant epilepsy, it is also an evaluated and promising treatment option for many psychiatric disorders, such as depression, dementia, schizophrenia, and somatoform disorder [69–72]. In the USA and Europe, VNS therapy is authorized as an adjunctive long-term treatment for TRD patients who have not responded to four different antidepressant treatments, indicating a more severe form of TRD [73].

VNS can be performed in two main ways: invasive and non-invasive. Invasive VNS (iVNS) involves surgically implanting a small pulse generator in the left thoracic region. Electrical leads are affixed to the left vagus nerve above its cardiac branch. The device delivers continuous stimulation, with adjustable parameters such as current, pulse width, and frequency [74]. Non-invasive VNS (tVNS) does not require surgery; instead, a stimulator attached to the auricular concha via ear clips delivers electrical impulses to the vagus nerve’s auricular branch [75, 76]. The most common acute complications from VNS include increased salivation, coughing, vocal cord paralysis, and lower face weakness. Less commonly, patients may experience bradycardia, and, rarely, asystole. In general, all side effects are easily reversible [77, 78].

For VNS, the critical pathway involves the tractus solitarius terminating in the nucleus tractus solitarius (NTS). Ascending NTS fibers project primarily to the pontine parabrachial nucleus and other regions, including the medullary and pontine nuclei, cerebellar areas, and periaqueductal gray [79]. Key NTS projections for TRD modulation target brainstem nuclei (medulla and pons) that regulate the secretion of biogenic amines associated with mood. The NTS sends projections to the pontine locus ceruleus, the main brainstem site for noradrenergic nuclei, as well as to the medullary and pontine raphe nuclei, the primary brainstem regions for serotonin nuclei [80]. Moreover, some NTS fibers bypass the NTS, sending projections to regions known to be involved in the development of MDD, including the hypothalamus, thalamus, nucleus accumbens, amygdala, and stria terminalis [81, 82].

Recent research provides promising evidence supporting the use of VNS as an adjunctive treatment for TRD. In a double-blind trial by Aaronson et al. involving 331 patients with TRD, adjunctive VNS was investigated at low (0.25 mA, 130 µs pulse width), medium (0.5–1.0 mA, 250 µs), and high (1.25–1.5 mA, 250 µs) current levels over the course of a year. VNS therapy was generally well tolerated across all patients. In the acute phase, all groups demonstrated statistically significant improvement. TRD patients who received VNS in addition to standard treatment showed significant improvement by the end of the study compared to their baseline condition, with these effects lasting for at least 1 year. Sustained response was more strongly associated with higher electrical dose settings—post hoc analyses revealed a statistically significant correlation between the total daily charge delivered and the reduction in depressive symptoms [83]. Another study highlighted the effectiveness of adjunctive VNS in improving outcomes for patients with TRD, in both ECT responders and non-responders [84]. The D-23 VNS registry included 489 patients treated with VNS and 276 patients receiving standard treatment. It revealed that patients receiving VNS had a significantly higher cumulative remission rate (43.3%) compared to those in the treatment-as-usual group (25.7%). The study also found that ECT responders who received VNS had a higher 5-year cumulative response rate (71.3%) compared to those in the treatment-as-usual group (56.9%). Even among ECT non-responders, VNS led to a better response rate (59.6%) compared to treatment-as-usual (34.1%). Additionally, VNS was associated with lower all-cause mortality and an anti-suicidal effect [83, 84].

 While many studies highlight VNS as an effective treatment for TRD, a systematic review by Lv et al. reported no significant antidepressant benefits [85]. It also emphasized the need for further research to fully evaluate its effectiveness and safety [85], indicating that this treatment still requires extensive assessment.

## Other non-invasive brain stimulation methods

Other non-invasive brain stimulation techniques include transcranial direct current stimulation (tDCS), transcranial alternating current stimulation (tACS), theta-burst stimulation (TBS), and random noise current stimulation (tRNS). tDCS works by applying a weak (0.5–2 mA) direct current via scalp electrodes [86]. Several tDCS studies have shown its efficacy, acceptability, and safety in the treatment of [86, 87]. However, it is not effective in TRD [88, 89].

tACS involves the delivery of alternating electric currents to the scalp. They have a sinusoidal waveform where the voltage changes gradually from positive to negative every half-cycle [90]. Due to the ability to noninvasively modulate brain oscillations, it has been investigated mainly in cognitive neuroscience [91]. The results of whether it is effective in depression are conflicting and the number of studies is too small to draw certain conclusions [92]. One protocol with positive results was gamma-tACS over F3/F4, which might be explained as targeting the hypoconnectivity in the dorsolateral prefrontal cortex and by extension the frontoparietal network [93]. Interestingly, the same settings were not effective in a single session, which suggests the superiority of multiple-session protocols.

TBS, particularly in its intermittent form (iTBS), is a novel and efficient variant of repetitive transcranial magnetic stimulation (rTMS). It delivers therapeutic magnetic pulses in a shorter duration—approximately 3 min compared to the standard 37.5 min for traditional rTMS [51]. A small number of sham-controlled trials, and one large comparative trial, support the efficacy of TBS, offering a time-efficient alternative to traditional rTMS protocols, not only in MDD, but also in TRD [94, 95]. However, more well-designed trials are needed to establish the optimal protocol settings. tRNS is a technique that delivers mild, randomly fluctuating electrical currents to the scalp. It has been mostly investigated in working memory and perception [96].

## Complementary medicine

An increasing number of patients struggling with TRD are seeking alternative and unconventional methods to manage their symptoms. Complementary and alternative medicine (CAM) therapies offer more than 120 health systems, practices, or products distinct from conventional medicine [97]. Nonetheless, few of them have sufficiently proven evidence to be considered effective in TRD therapy. Those CAM therapies of promising efficacy include exercise, light therapy, yoga as well as mindfulness-based cognitive therapy, and natural health supplements such as omega-3 fatty acids and S-adenosylmethionine.

Exercise is proven to exert a significant impact on mental health among patients suffering from MDD. However, its efficacy in TRD treatment needs to be evaluated in future studies, as there is a limited number of studies covering this area. Although there are no specific guidelines for physical activity in TRD, it appears beneficial for patients to exercise 2–5 times per week for 30–60 min complementarily to pharmacotherapy [98].

Light therapy is based on daily exposure to artificial bright light which seems to be an effective adjunctive treatment if combined with antidepressant medications. Photomodulation (PBM) refers to the utilization of red or near-infrared light by placing light sources on the head with the aim of stimulating a specific cerebral area [99]. It has been shown to positively impact working memory, cognitive inhibition, cerebral blood flow, and brain metabolic activity [100, 101]. Various studies support PBM’s ability to reduce depressive symptoms [102–104]. Future research is needed to establish a minimum effective dose and treatment protocols [105]. Notwithstanding preliminary evidence for the efficacy of light therapy in MDD treatment, further research is needed to confirm the same outcomes for TRD patients.

Yoga is a combination of breathing practices, meditation, and body movement which antidepressant effects seem apparent yet not fully understood. Although its beneficial impact on depressive symptoms has been confirmed, there is limited data considering yoga efficacy in TRD [106].

Mindfulness-based cognitive therapy incorporates elements of both mindfulness meditation and cognitive–behavioral therapy. Originally, the intervention was conceived to prevent relapses of recurrent MDD. The therapy demonstrates effectiveness in chronic depression as well as promising results in TRD treatment; however, the evidence considering the second one is preliminary.

Dietary supplements such as omega-3 fatty acids and S-adenosylmethionine both exhibit promising efficacy in TRD with moderately mild adverse effects [107]. A study by Krawczyk et al. showed that augmenting a standardized antidepressant treatment with omega-3 fatty acids resulted in a marked improvement in depression symptoms in the majority of patients with TRD. Clinical improvement was proportional to the one achieved in the control group by potentiating therapeutic effects with lithium and lamotrigine, without significant side [108].

## Treatment-resistant depression in geriatric patients

Depression among older adults is a significant healthcare concern. While seniors generally have lower overall rates of depression compared to middle-aged adults, geriatric patients who are chronically ill or disabled experience a significantly higher prevalence [109]. Notably, 10–12% of elderly hospitalized patients and 12–14% of nursing home residents suffer from MDD [110]. Most studies indicate that late-life depression (LLD) compared to early-onset depression, is accompanied by more comorbidities, higher mortality rates, and greater levels of disability [109]. LLD is often linked to cognitive impairment, which may be partially explained by various overlapping pathophysiological factors, such as vascular risk or present neuroinflammation [111]. In a study by Lu et al., a longer duration of depression was associated with lower cognitive scores among geriatric patients [112]. The co-occurrence of neurodegenerative processes and depression can significantly impact treatment outcomes. While antidepressant medications generally exhibit limited efficacy in LDD, patients with structural brain abnormalities and cognitive impairment demonstrate an especially poor therapeutic response [113, 114].

Geriatric TRD exact prevalence is uncertain, but estimates range from 18 to 40% [115]. Older adults with TRD require particular attention—clinicians should first assess the patient’s adherence since it is the leading cause of MRD among the elderly [116]. Among the available pharmacotherapies, the most effective one appears to be augmentation with aripiprazole or bupropion—it is associated with a statistically significant improvement in psychological well-being [117]. Other promising therapies include rTMS, sequential bilateral theta burst stimulation, and cognitive remediation [118].

## Conclusions

New pharmacological approaches to TRD involve glutamatergic modulators like ketamine and esketamine, which are effective but may have varying tolerability. Additionally, psilocybin and anti-inflammatory drugs are being explored for their potential benefits, though further research is needed to confirm their efficacy and safety. ECT demonstrates a high response rate in acute treatment and remains an important component of TRD treatment in cases of non-response to standard drug therapy and psychotherapy. Ongoing refinement of the test’s parameter selection aims to enhance the effectiveness of long-term treatment and reduce the risk of relapse while minimizing the procedure’s side effects.

TMS is a non-invasive treatment which stimulate prefrontal cortex areas crucial in the development of depression. Its efficacy depends on many factors and even though it is less invasive than ECT as well as safe and recommended in TRD treatment, additional research on its long-term effects and maintenance protocols is essential.

DBS offers hope as a treatment method for patients with TRD, having therapeutic potential in cases where traditional therapies have been ineffective. However, further research is crucial to optimize its efficacy.

VNS is a promising adjunctive treatment for TRD, showing significant improvements in depressive symptoms, especially at higher electrical doses, with evidence suggesting better outcomes in both ECT responders and non-responders. Nonetheless, despite positive findings in various studies, recent meta-analyses indicate the need for further research to fully validate its antidepressant efficacy and safety.

CAM therapies are diversified and accessible for many patients with MDD who often benefit from such adjunctive treatment. Although the majority of such therapies are not evaluated, some of them such as exercise, yoga, mindfulness-based cognitive therapy or omega-3 fatty acids, and S-adenosylmethionine supplementation exhibit efficacy when combined with pharmacotherapy. Notwithstanding, due to the lack of evidence for many CAM therapy efficacy and safety, further research should be conducted.

Novel therapies for TRD show great promise; however, they are not effective in all patients and require further investigation.
